# Increased Risk for Congenital Heart Defects in Children Carrying the ABCB1 Gene C3435T Polymorphism and Maternal Periconceptional Toxicants Exposure

**DOI:** 10.1371/journal.pone.0068807

**Published:** 2013-07-17

**Authors:** Chuan Wang, Liang Xie, Kaiyu Zhou, Yalan Zhan, Yifei Li, Huaying Li, Lina Qiao, Fang Wang, Yimin Hua

**Affiliations:** 1 Department of Pediatric Cardiovascular Disease, West China Second University Hospital, Sichuan University, Chengdu, Sichuan, China; 2 The Pulmonary Vascular Remodeling Research Unit, West China Institute of Women and Children’s Health, West China Second University Hospital, Sichuan University, Chengdu, Sichuan, China; 3 West China Medical School of Sichuan University, Chengdu, Sichuan, China; 4 Key Laboratory of Obstetric and Gynecologic and Pediatric Diseases and Birth Defects of Ministry of Education, West China Second University Hospital, Sichuan University, Chengdu, Sichuan, China; Tabriz University of Medical Sciences, Iran (Republic of Islamic)

## Abstract

**Backgrounds:**

The etiology of congenital heart defect (CHD) is commonly believed to involve the interaction of multiple environmental and genetic factors. This study aimed to explore the joint effects of the ABCB1 gene C3435T polymorphism and maternal periconceptional toxicants exposure on the CHD risk in a Han Chinese population.

**Methods:**

An age and gender matched case-control study with standardized data collection involving 201 pairs was conducted. Periconceptional toxicants exposure was obtained through a structured questionnaire. A job exposure matrix (JEM) was used for toxicants exposure assessment. Genotyping of the ABCB1 C3435T polymorphism was performed by sequencing. Logistic regression analysis was performed to assess the joint effects of the ABCB1 gene C3435T polymorphism and toxicants exposure on the risk of CHD. Placenta tissues and umbilical cords were collected to investigate the impact of C3435T polymorphism on the transcription and translation activities of ABCB1 gene.

**Results:**

Maternal periconceptional exposures to phthalates (adjusted OR: 1.6; 95%CI: 1.0–2.6) and alkylphenolic compounds (adjusted OR:1.8; 95%CI:1.1–3.0) were associated with a higher incidence of CHDs in general. More cases were carriers of the ABCB1 CC/CT genotypes (OR: 2.0, 95%CI: 1.1–3.5, P-value: 0.021). Children carrying the CC/CT genotype and periconceptionally exposed to phthalates and alkylphenolic compounds suffered almost 3.5-fold increased risk of having CHD than non-exposed children with TT genotype (adjusted OR: 3.5, 95%CI: 1.5–7.9, P-value: 0.003), and the OR changed to 4.4 for septal defects (adjusted OR: 4.4,95%CI:1.8–10.9,P-value:0.001). The ABCB1 mRNA expression of the TT genotype was significantly higher than that of the CC genotype (P = 0.03). Compared with TT genotype, lower P-glycoprotein expression was observed for the CC/CT genotypes.

**Conclusion:**

The C3435T polymorphism in the ABCB1 gene of fetus increases the risks of CHD in a Han Chinese population when the mothers are exposed to phthalates and alkylphenolic compounds during the periconceptional period, particularly for septal defects.

## Introduction

Worldwide congenital heart defect (CHD) is one of the most common birth defects, occurring in 7 to 8 per 1000 live births in China. After years of study, the etiology of CHD is still unclear, but is commonly believed to involve the interaction of multiple environmental and genetic factors [1], [2]. Substantial epidemiological data have demonstrated that the exposure to several toxicants during the periconceptional period was associated with an increased risk of CHD [3], [4], [5], [6]. Nevertheless, not all women exposed to toxicants during the periconceptional period give birth to a child with CHD. The reasons for such phenomenon still remain elusive.

There is increasing evidence that the placenta expresses a range of transporters capable of controlling the transplacental disposition of many toxicant agents and thereby playing a crucial role in fetal protection against maternal toxins [7], [8], [9], [10], [11], [12], [13]. Of main interest are the ATP-binding cassette (ABC) transporters, particularly the first discovered and so far the best characterized of drug efflux transporters, P-glycoprotein (P-gp), encoded by the ABCB1 or MDR1 gene in human [14].

Specially locating in the maternal-facing apical membrane of the syncytiotrophoblast, together with its vectorial transport capacity, P-gp has the capacity to actively efflux a wide range of toxicants back to the maternal circulation, thus preventing potentially harmful compounds from entering the fetal compartment [7], [14], [15], [16], [17]. The contribution that P-gp makes to the barrier function of the placenta is best illustrated in the ABCB1 knock-out mice. Studies in these animals have shown that treatment of heterozygous females with an isomer of avermectin, a known teratogenic substrate of P-gp, results in 100% cleft palate in fetuses deficient in P-gp(−/−), whereas the heterozygotes(+/−) are less susceptible and those expressing abundant P-gp(+/+) are protected from the effects of avermectin at the doses studied [18]. From this background, the differences in expression of P-gp in placenta may play a significant role in determining the susceptibility to chemically induced birth defects. Moreover, studies have illustrated that the expression of P-gp was significantly highest in the first trimester [19], [20], [21], which is in accordance with the critical period of cardiac development. Taken together, the interindividual variability in expression of P-gp in placenta is likely to be associated with fetal susceptibility to toxicants induced CHD.

To date, evidence has suggested that several single nucleotide polymorphisms (SNPs) in ABCB1 gene were associated with altered P-gp expression and transport activity in placenta [22], [23]. Of which, a synonymous mutation in exon 26 (C3435T), is the most commonly studied one. Because the placenta is of fetal origin, this polymorphism in fetus may modify the interindividual susceptibility to CHD when the fetus is exposed to toxicants during the periconceptional period. Therefore, a case-control study was conducted to explore the joint effects of the ABCB1 C3435T polymorphism and maternal toxicants exposure during the periconceptional period on CHD risk in a Han Chinese population. Furthermore, maternal placentas and umbilical cords were also collected to examine the effect of the C3435T polymorphism on the ABCB1 gene transcription and protein expression.

## Materials and Methods

### Ethics Statements

Informed written consent was obtained from the parents, who were on the behalf of their child as well; about their participation in the study after the nature of this study has been fully explained to them. Informed written consent was also obtained from the mothers when we collected the placenta tissue and umbilical cords. The study was approved by the University Committee on Human Subjects at Sichuan University.

### Study Participants

An age and gender matched case-control study (1∶1) was conducted from March to December in 2012. 216 children with CHD and 216 children without any congenital malformations or chromosomal abnormalities were enrolled. All children were recruited from the Department of Pediatric Cardiology in West China Second University Hospital, Sichuan University and born in Sichuan province and their birth mothers were local residents. When a new case was confirmed, we downloaded all information on eligible children from the hospital information system, and then the corresponding control was randomly selected from system matching with age and gender. The diagnoses of all cases were confirmed by cardiac catheterization and the diagnoses of all controls were checked by experienced pediatric cardiologists according to medical records. To minimize recall bias of exposure by the mother to the greatest extent, all cases and controls recruited were younger than 3 years old. We excluded those whose birth mother was absent or rejected to be interviewed. Additionally, cases with a family history of CHD and mothers with pre-gestational diabetes were also excluded, as these are strong risk factors for CHD. After the exclusion, 201 pairs were used for analysis. The included CHD phenotypes were perimembranous septal defect (pVSD, n = 125), atrial septal defect (ASD, n = 41), pulmonary valve stenosis (PS, n = 22), Tetralogy of Fallot (TOF, n = 5), pulmonary atresia with ventricular septal defect (PA/VSD, n = 2), anomalous pulmonary venous connection (APVC, n = 2), atrioventricular septal defect (AVSD, n = 2), transposition of the great arteries (TGA, n = 1),and truncus arteriosus (n = 1).

### Placenta Collection

Thirty-five human placentas and umbilical cords were consecutively obtained from mothers who delivered at the Department of Gynecology and Obstetrics in West China Second University Hospital, Sichuan University by a well-trained nurse. The tissues were immediately frozen in liquid n**i**trogen and stored at −80°C until use. Moreover, the maternal and neonatal clinical data were also collected, including maternal age, maternal ethnicity, health conditions, pregnancy complications, any medication consumptions, and neonatal birth outcomes (eg. sex, gestational duration, birth length, and birth weight).

Only placentas and umbilical cords from healthy Han Chinese mothers with uncomplicated pregnancy and infants with a gestational duration between 38–41 weeks and a birth weight between 2500–4000 g were included. Those from mothers with chronic diseases(eg. Hepatitis, hypothyroidism, polycystic ovary syndrome, and sexually transmitted infections), pregnancy complications (eg.gestational diabetes mellitus, pregnancy-induced hypertension, intrahepatic cholestasis of pregnancy, and placentaprevia), and any medication use were excluded.

### Data Collection and Quality Control

Structured questionnaires with pre-coded and open-ended questions were used for data collection. All questionnaires were pre-tested and revised accordingly. The questionnaires survey was conducted when the children were admitted to the hospital and their birth parents were interviewed face to face by a well trained interviewer, who was blinded to the participant’s group assignment. The questionnaires were double checked to assure its completeness and consistency. All the participants’ birth parents were retrospectively interviewed in a similar manner during the period of the study.

The contents of the questionnaire included:

1. Personal characteristics: child’s name, gender, race, birth date, address, parents’ education, and yearly income per person.
2. Conditions of participant’s birth: gestational weeks, birth length, birth weight, birth asphyxia or hypoxia, and family history of CHD or other congenital malformations.
3. Characteristics of parents: age at pregnancy, pre-pregnancy weight and height, occupational exposure history, disease history, and lifestyle (smoking, consumption of alcohol, tea, and coffee).
4. Conditions of pregnancy: reproductive history, artificial fertilization or not, folic acid supplementation, diseases, medication use during pregnancy, and other potential environmental toxicants exposure (e.g. house decoration during the periconceptional period).

### Toxicants Exposure Assessment during the Periconceptional Period

In the questionnaire, each mother was asked to report whether she worked in a full-time or part-time job for at least 1 month in duration during the periconceptional period. If she did report working, she was asked a series of questions about each job she held, including the job title, main tasks, and duties. Beginning and ending dates for each job were collected, as well as the work hours per day and work days per week. We assessed occupational exposure to chemicals by applying a job exposure matrix (JEM), with a focus on endocrine-disrupting chemicals [24], [25]. Job descriptions were coded into job titles by the Dutch Standard Classification of Occupations, and linked to the JEM, which was based on the judgment of occupational hygienists who estimated for particular jobs the likelihood of exposure to seven categories of chemicals, namely pesticides, polychlorinated compounds, phthalates, bisphenol A, alkylphenolic compounds, heavy metals, and miscellaneous agents. Since there might be some differences in toxicants exposure for the same occupation between Western Europe and China, the original JEM served as a starting point or reference material and three Chinese industrial hygienists from School of Public Health, Sichuan University, were asked to make adjustments for exposure assessment based on their knowledge of tasks and work environment of various occupations in China. Finally, exposure probability scores were adjusted based on the consensus judgments of these three experts. Exposure assessment by the JEM was blinded to the genotyping outcome and to the participant’s group assignment. The JEM focuses on the most important chemicals with relevant exposures in the occupational setting. The occupational hygienists scored the probability of exposure to each chemical group for all job titles, in three levels: ‘unlikely’ (0), ‘possible’ (1) and ‘probable’ (2). For this study we emerge categories 1 and 2 into one category indicating the possible occurrence (yes/no) of exposure to chemicals. An overall classification of “exposure to one of these six exposure categories ’ was collated if this category was scored as ‘yes’ by the JEM according to the job description and work status. That is to say, one individual can be included in two or more exposure categories. A mother was considered exposed if she reported having one or more jobs at least for 1 month in duration during the periconceptional period that was rated as exposed by this method. Meanwhile, a mother was considered unexposed if all her jobs during this time period was rated as unexposed to all the evaluated chemicals by this method or if she was employed exclusively outside the periconceptional period.

Environmental exposure only included living in a newly decorated house for at least 1 month during the periconceptional period. Two studies have confirmed that higher concentrations of phthalates and alkylphenolic compounds were present in newly decorated apartments [26], [27]. Thus living in a newly decorated house during the periconceptional period was defined as the likelihood of exposure to phthalates and alkylphenolic compounds.

Given the relatively small number of exposed parents in any particular environmental and occupational category, statistical analysis for toxicant exposure was performed including both environmental and occupational exposure.

### Genotyping

DNA was extracted from leucocytes of the whole blood and from the umbilical cord by a commercial SE blood DNA isolation kit (D3471-02, Omega Bio-tek, USA) and a commercial tissue DNA isolation kit (D3396-01, Omega Bio-teck,USA) respectively. A 110-bp fragment was amplified using 0.25 ul Taq DNA polymerase (Takara), 2.5 ul 10×PCR buffer,1 ul dNTP(2.5 mmol L^−1^),0.5 ul forward primer (5′-GAGCCCATCCTGTTTGACTGC-3′, 10 pmol L^−1^),0.5 ul reverse primer(5′- TGTATGTTGGCCTCCTTTGCTG-3′,10 pmol lL**^–^** ^1^), and 30 ng of genomic DNA in a total volume of 25 ul. PCR conditions were 34 cycles of 30 sec at 95°C, 30 sec at 63°C, and 1 min at 72°C, preceded by an initial denaturation of 5 min at 95°C, and followed by a final extension of 7 min at 72°C. PCR products were purified using a Watson DNA Purification Kit (Watson Company, Shanghai, China) and were directly sequenced on an ABI 3730 (AppliedBiosystems, Life technologies, Foster City, CA) according to the manufacturer’s protocol.

### RNA Extraction and cDNA Synthesis

Total RNA was extracted from 50–100 mg of frozen placental tissue by TRIzol (Invitrogen, Life technologies, Carlsbad, CA) and subjected to qualitative and quantitative measurements using a spectrophotometer (GeneQuant 100, GE healthcare). 1 ug of total extracted RNA was reverse transcribed to cDNA using Prime Script RT reagent kit with gDNA eraser (DRR047A, Takara, Dalian, China) according to the manufacturer’s instructions.

### Real-time Quantitative Polymerase Chain Reaction (qPCR)

Real-time RT-PCR was performed with cDNA templates and SsoFast EvaGreen Supermixture (Bio-Rad Laboratories, Hercules, CA). Briefly, sequences were amplified using 5 ul reaction mixture, 0.3 ul forward primer (10 pmolL^−1^), 0.3 ul reverse primer (10 pmol lL^–1^), 3.4 ul nuclease-free H_2_O, and 1 ul cDNA in a total volume of 10 ul. PCR conditions were 39 cycles of 30 sec at 95°C, 30 sec at 60°C, and 1 min at 72°C, preceded by an initial denaturation of 3 min at 95°C, and followed by a continuous melt curve from 65–95°C. All the primer sets were tested to ensure the efficiency of amplification over a range of template concentrations. All samples were amplified in triplicates. Expression level of ABCB1 transcript was represented by the mean of triple tests. The relative expressions of ABCB1 mRNA were normalized to expression of GAPDH using 2^−△△Ct^ method.

The primer sequences used were as follows:

5′- TCCTGGAGCGGTTCTACGAC-3′ (ABCB1-F),

5′-GCTGCAGTCAAACAGGATGG-3′(ABCB1-R),

5′-GAAGGTGAAGGTCGGAGTC-3′(GAPDH-F),

5′-GAAGATGGTGATGGGATTTC-3′(GAPDH-R).

### Western Blot Analysis of P-glycoprotein

100 mg placenta tissues were homogenized in buffer containing 1 ml RIPA(P0013B,Beyotime, China) and 10 ul complete protease inhibitor cocktail (P8340, Sigma-Aldrich). The homogenate was then centrifuged at 12,000 g for 5 min at 4°C. Protein concentration was determined using enhanced BCA protein assay kit (P0010S, Beyotime, China) according to the manufacturer’s protocol. Total protein (30 ug/lane) was separated on 8% SDS-polyacrylamide gel and transferred to polyvinylidene fluoride (PVDF) membranes (Millipore, Bedford, MA). The membranes were blocked in 5% BSA in Phosphate-buffered saline containing 0.1% Tween-20 (PBST). Thereafter, blocked membranes were incubated with overnight at 4°C with monoclonal primary antibody for P-gp (C219, No.517310, Calbiochem, 1∶50,) and a-tublin (ab6160, Abcam, 1∶5000). Following extensive washing with PBST, membranes were incubated with a 1∶10000 dilution of horseradish peroxidase-conjugated goat anti-mouse immunoglobulin G (IgG) secondary antibodies (No.107724,ZSGB-BIO,China). After extensive washing with PBST, protein-antibody complexes were visualized by the enhanced chemiluminescene detection system.

### Statistical Analysis

All materials were coded and data was doubly inputted using EPIDATA 3.1 version and all analysis were conducted with SPSS 17.0 version. For data analysis, BMI was calculated as weight divided by height (m) squared and it was divided into 4 categories: underweight (<18.5 Kg/m^2^), normal (18.5–23.9 Kg/m^2^), overweight (24.0–27.9 Kg/m^2^) and obese (≥28 Kg/m^2^), in accordance with the criteria specially formulated for Chinese [28], [29]. The periconceptional period was defined as 4 weeks prior to conception until the end of first trimester. The maternal use of folic acid in the periconceptional period was defined as the daily use of at least 400 ug folic acid during the complete periconceptional period. All others were classified as non-users. We defined smoking, alcohol consumption, tea-drinking, coffee-drinking, and medication use as any use during the periconceptional period.

For the epidemiological data analysis, firstly, socio-demographic and lifestyle characteristics of children, mothers, and fathers were compared respectively between the groups using Mann-Whitney U-test for quantitative variables and Chi–Square test for categorical variables. Fisher’s Exact Test was also used if not matched.Then, multivariate conditional stepwise regression analysis was performed to estimate the odds ratios (ORs) and 95% confidence intervals (CIs) for the associations between maternal toxicants exposure and CHDs. The ORs were adjusted for the following potential confounding factors: maternal age at births, maternal education level, BMI, gravity, history of previous abortions, periconceptional use of folic acid, medication use, settlement, and paternal toxicants exposure.

In the genetic study, the chi-square test was used to test for deviation from Hardy–Weinberg equilibrium and to compare allelic and genotypic frequencies between the groups. For SNP alleles, we calculated three categories of p values for the different probable function of the mutant allele as follows: dominant, recessive, and co-dominant. The results showed that maternal exposure to phthalates (adjusted OR: 1.6; 95%CI: 1.0–2.6) and alkylphenolic compounds (adjusted OR: 1.8; 95%CI: 1.1–3.0) was associated with a higher incidence of CHDs in general. In light of these findings, we defined the “toxicant exposure” as maternal periconceptionally exposure to phathalates and/or alkyophenolic compounds in the present study. In the end, logistic regression analysis was performed to assess the joint effects of the ABCB1 gene C3435T polymorphism and “toxicants exposure” on the risk of CHDs. We coded separate categories for the risk of genotype in combination with periconceptional toxicants exposure. ABCB1 TT carriers without periconceptional toxicants exposure were expected to have the lowest risk and therefore considered as the reference category. Whereas the highest risk group consisted of ABCB1 CC/CT carriers with periconceptional toxicants exposure. In addition, the ORs were also adjusted for confounding factors mentioned above. Moreover, the genotype distribution and allele frequencies between cases and controls restricted to “toxicants exposure” were also calculated.

For the ABCB1 mRNA expression in the placenta, data was expressed as as mean±SEM. Differences among three genotypes were determined by one-way ANOVA followed by a Student-Newman-Keuls multiple comparison post hoc tests.

Power of this study was calculated by Quanto v1.2.4 [30], suggesting that the sample size of this study could detect an OR of 1.53 and 2.42 for genetic study and gene-environment study, respectively. A 2-tailed p value <0.05 was chosen as the level of significance. Given that only 22 patients with pulmonary valve stenosis (PS) and 13 patients with complex congenital heart defects were recruited in the present study, statistical analysis of these two subgroups might not be feasible due to the limited sample size. Therefore, stratified analysis of the different CHD phenotypes was only conducted for septal defects.

## Results

Sociodemographic and lifestyle characteristics of mothers, fathers, and children are illustrated in table 1 in detail. The patients showed a significantly lower birth weight after adjustment for gestational age, compared with the control children (P = 0.013). In addition, cases and controls were significantly different for maternal education level (P<0.001), paternal education level (P = 0.002) and maternal medication use during the periconceptional period (P = 0.001).

**Table 1:** Sociodemographic and lifestyle characteristics of mothers, fathers and children.

Variables	Cases^a^, *n* = 201	Controls^a^, *n* = 201	P-value b
**Characteristics of children**			
Age at in take (months)	26.20±7.30	26.10±7.20	
Gender (Male)	89 (44.3)	89 (44.3)	
Gestational age			0.512
>42 weeks	2(1.0)	4(2.0)	
37–42 weeks	188(93.5)	182(90.5)	
<37 weeks	11(5.5)	15(7.5)	
Birth weight (Kg)	3.13±0.48	3.25±0.58	0.013
Birth length (cm)	49.86±2.10	50.10±2.10	0.245
Twins (yes)	8(4.0)	6(3.0)	0.787
Artificial fertilization (yes)	10(5.0)	5(2.5)	0.188
**Characteristics of mothers**			
Age at births (years)			0.345
≥35	16(8.0)	12(6.0)	
20–35	161(80.1)	172(85.6)	
≤20	24(11.9)	17(8.5)	
Education level			<0.001
illiteracy	1(0.5)	8(4.0)	
primary school	27(13.4)	23(11.4)	
middle school	88(43.8)	48(23.9)	
high school	45(22.4)	47(23.4)	
college or higher	40(19.9)	75(37.3)	
Body mass index(kg/m^2^)^c^			0.786
underweight	43(21.4)	38(18.9)	
normal	145(72.1)	148(73.6)	
overweight	13(6.5)	15(7.5)	
Gravity			0.468
1	67(33.3)	80(39.8)	
2	59(29.4)	59(29.4)	
3	41(20.4)	36(17.9)	
≥4	34(17.0)	26(13.0)	
History of previous abortions			
Spontaneous abortion	15(7.5%)	11(5.5)	0.417
Artificial abortion			0.218
0	99(49.3)	117(58.2)	
1	53(26.4)	46(22.9)	
2	36(17.9)	24(11.9)	
≥3	13(6.5)	14(7.0)	
Previous stillbirth (yes)	6(3.0)	3(1.5)	0.503
Ectopic pregnancy (yes)	4(2.0)	2(1.0)	0.685
Preconceptional d			
Folic acid (yes)	25(12.4)	31(15.4)	0.387
Smoking (yes)	9(4.5)	8(4.0)	0.804
Alcohol consumption(yes)	14(7.0)	18(9.0)	0.461
Tea-drinking (yes)	21(10.4)	21(10.4)	1.000
Coffee-drinking (yes)	6(3.0)	5(2.5)	0.760
Medication use (yes)	78(38.8)	48(24.0)	0.001
Settlement			0.203
urban	90(44.8)	107(53.2)	
rural	80(39.8)	71(35.3)	
suburban	31(15.4)	23(11.4)	
**Characteristics of fathers**			
Age at births (years)			0.692
≥35	36(17.9)	33(16.4)	
20–35	164(81.6)	168(83.6)	
≤20	1(0.5)	0(0.0)	
Education level			0.002
illiteracy	2(1.0)	4(2.0)	
primary school	22(10.9)	19(9.5)	
middle school	90(44.8)	59(29.4)	
high school	41(20.4)	40(19.9)	
college or higher	46(22.9)	79(39.3)	
Smoking (yes)	79(40.5)	76(38.2)	0.637
Alcohol consumption(yes)	116(59.5)	109(54.8)	0.345
Paternal toxicants exposure(yes)	66(32.8)	47(23.4)	0.035

a Quantitative data and categorical data were expressed as mean ± SD and n (%), respectively.

b Mann-Whitney U test was used to compare means; chi-square test was used to compare proportions and Fisher’s Exact Test was also used if not matched. A 2-tailed p value was chosen as the level of significance.

c Body mass index(BMI) was calculated as weight divided by height (m) squared and it was divided into 4 categories: underweight (<18.5 Kg/m2), normal (18.5–23.9 Kg/m2), overweight (24.0–27.9 Kg/m2) and obese ?≥?? Kg/m2), in accordance with the criteria specially formulated for Chinese [28], [29].

d The periconceptional period was defined as 4 weeks prior to conception until the end of first trimester. The maternal use of folic acid in the periconceptional period was defined as the daily use of at least 400 ug folic acid during the complete periconceptional period. In consistent users were classified as non-users. We defined smoking, alcohol consumption, tea-drinking, coffee-drinking and medication use as any use during the periconceptional period.

Table 2 presents the associations between maternal periconceptional occupational exposures and CHDs/septal defects. After adjusting for confounding factors mentioned in the statistical analysis, the results showed that both maternal periconceptionally exposures to phthalates (adjusted OR: 1.6; 95%CI: 1.0–2.6) and alkylphenolic compounds (adjusted OR: 1.8; 95%CI: 1.1–3.0) were associated with a higher incidence of CHDs in general. The significant associations were also found for septal defects (phthalates: adjusted OR: 1.9; 95%CI:1.1–3.3; alkylphenolic compounds: adjusted OR: 1.9; 95%CI:1.1–3.3).

**Table 2:** Associations between maternal periconceptional occupational exposures and CHDs/septal defects in detail.

Exposure categories	Controls(%)N = 201	Cases(%) N = 201	OR(95%CI)crude	OR(95%CI)adjusted	Septal defect (%) N = 166	OR(95%CI)crude	OR(95%CI)adjusted a
Pesticides	17(8.5)	13(6.5)	0.7(0.4–1.6)	0.8(0.3–1.9)	10(6.0)	0.7(0.3–1.6)	0.8(0.3–2.0)
Polychlorinated compounds	7(3.5)	11(5.5)	1.6(0.6–4.2)	1.3(0.5–3.7)	11(6.6)	2.0(0.7–5.2)	1.6(0.5–4.6)
Phthalates	40(19.9)	59(29.4)	1.7(1.1–2.7)	1.6(1.0–2.6)	55(33.1)	2.0(1.2–3.2)	1.9(1.1–3.3)
Alkylphenolic compounds	44(21.9)	66(32.8)	1.7(1.1–2.7)	1.8(1.1–3.0)	54(32.5)	1.7 (1.1–2.7)	1.9(1.1–3.3)
Bisphenol A	3(1.5)	4(2.0)	1.3(0.3–6.1)	1.2(0.2–6.0)	4(2.4)	1.6(0.4–7.4)	1.4(0.3–7.0)
Heavy metals	24(11.9)	35(17.4)	1.6(0.9–2.7)	1.4(0.8–2.6)	34(20.5)	1.9(1.1–3.4)	1.6(0.9–3.0)
Toxicants exposures^b^	85(42.3)	54(26.9)	2.0(1.3–3.0)	2.0(1.2–3.2)	72(43.4)	2.1(1.3–3.2)	2.1(1.3–3.6)

CI: confidence interval; OR: odds ratio.

a risk estimates adjusted for maternal age at births, maternal education level, BMI, gravity, history of previous abortions, periconceptional use of folic acid, medication use,settlement and paternal toxicants exposure.

b “toxicants exposures” was defined as maternal periconceptionally exposure(s) to phathalates and/or alkyophenolic compounds.

The genotype distributions and allele frequencies of C3435T were listed in table 3.The genotype distributions were in Hardy-Weinberg equilibrium and significantly different between cases and controls (P = 0.043). In the dominant model, more cases were carriers of the ABCB1 CC/CT genotypes, which were significantly associated with an increased risk of CHD (OR: 2.0, 95%CI: 1.1–3.5, P-value: 0.021). Meanwhile, allele frequencies were also significantly different between cases and groups (OR: 1.4, 95%CI: 1.1–1.9, P-value: 0.016). However, there was no significant difference when applying the recessive model.

**Table 3:** The genotype distribution and allele frequencies of C3435T.

Genotyping distribution	Control (%)	Cases (%)	P-value	OR (95%CI)	Septal defects (n/%)	P-value	OR (95%CI)
C/C	69(34.3)	86(42.8)	0.012	2.2(1.2–4.2)	75(45.2)	0.006	2.6(1.3–5.2)
C/T	96(47.8)	95(47.3)	0.066	1.8(0.9–3.3)	76(45.8)	0.062	1.9(0.9–3.7)
T/T	36(17.9)	20(10.0)	0.043	reference	15(9.0)	0.021	reference
Hardy-Weinberg equilibrium P-value	P>0.05	P>0.05			P>0.05		
Dominant model							
C/C or C/T	165(82.1)	181(90.0)	0.021	2.0(1.1–3.5)	151(91.0)	0.014	2.2(1.2–4.2)
T/T	36(17.9)	20(10.0)			15(9.0)		
Recessive model							
C/C	69(34.3)	86(42.8)	0.082	1.4(0.9–2.1)	75(45.2)	0.034	1.6(1.0–2.4)
C/T or T/T	96(47.8)	115(57.3)			91(54.8)		
Codominant model							
C allele	234(58.2)	267(66.4)	0.016	1.4(1.1–1.9)	236(69.0)	0.002	1.6(1.2–2.2)
T allele	168(41.8)	135(33.6)			106(31.0)		

CI: confidence interval; OR: odds ratio.

The results of the interaction analysis of ABCB1 gene C3435T in conjunction with the maternal phthalates and/or alkylphenolic compounds exposure were shown in table 4. Children who carry the CC/CT genotype and have periconceptionally exposed to phthalates and/or alkylphenolic compounds have an almost 3.5-fold significantly increased risk of having CHD than non-exposed children with TT genotype (adjusted OR: 3.5, 95%CI: 1.5–7.9, P-value: 0.003). The OR changed to 4.4 for septal defects (adjusted OR: 4.4, 95%CI: 1.8–10.9,P-value:0.001).

**Table 4:** The results of the interaction analysis of ABCB1 gene C3435T polymorphism in conjunction with maternal phthalates and alkylphenolic compounds exposure.

*ABCB1 C3435T*	*Toxicants* *Exposure*	*Controls* *N = 201*	*Cases N = 201*	*OR(95%CI) crude*	*OR(95%CI) adjusted^a^*	*Septal defects N = 166*	*OR(95%CI) crude*	*OR(95%CI) adjusted^a^*
CC and C/T	Yes	43	79	3.3(1.5–7.0)	3.5(1.5–7.9)	67	3.9(1.7–8.9)	4.4(1.8–10.9)
	No	122	102	1.5(0.7–3.0)	1.6(0.8–3.4)	84	1.7(0.8–3.8)	1.9(0.8–4.3)
TT	Yes	11	6	1.0(0.3–3.2)	0.9(0.3–3.3)	5	1.1(0.3–4.1)	1.1(0.3–4.4)
	No	25	14	1.0(reference)	1.0(reference)	10	1.0(reference)	1.0(reference)
			P-value	0.003	0.003		0.003	0.001

CI: confidence interval; OR: odds ratio.

a Risk estimates adjusted for maternal age at births, maternal education level, BMI, gravity, history of previous abortions, periconceptional use of folic acid, medication use,settlement and paternal toxicants exposure.

Table 5 showed the genotype distributions and allele frequencies of C3435T restricted to phthalates and alkylphenolic compounds exposures. The results showed that the associations were stronger both for CHDs in general (OR: 3.4, 95%CI: 1.2–9.7, P-value: 0.020) and for septal defects (OR: 3.4, 95%CI: 1.1–10.5, P-value: 0.020) when applying the dominant model.

**Table 5:** The genotype distribution and allele frequencies of C3435T restricted to phthalates and alkylphenolic compounds exposures.

Genotyping distribution	Control (%)	CHD (%)	P-value	OR (95%CI)	Septal defect (%)	P-value	OR (95%CI)
C/C	16(29.6)	43(50.6)	0.017	4.9(1.6–15.5)	38(52.8)	0.007	5.2(1.6–17.5)
C/T	27(50.0)	36(42.4)	0.115	2.4(0.8–7.4)	29(40.3)	0.153	2.4(0.7–7.7)
T/T	11(20.4)	6(7.1)	0.017	reference	5(6.9)	0.015	reference
Dominant model							
C/C or C/T	43(79.6)	79(92.9)	0.020	3.4(1.2–9.7)	67(93.1)	0.020	3.4(1.1–10.5)
T/T	11(20.4)	6(7.1)			5(6.9)		
Recessive model							
C/C	16(29.6)	43(50.6)	0.015	2.4(1.2–5.0)	38(52.8)	0.009	2.7(1.3–5.6)
C/T or T/T	38(70.4)	42(49.5)			34(47.2)		
Codominant model							
C allele	59(71.8)	122(54.6)	0.003	2.1(1.3–3.5)	105(72.9)	0.003	2.2(1.3–3.8)
T allele	49(28.2)	48(45.4)			39(27.1)		

CI: confidence interval; OR: odds ratio.

**Figure 1:**
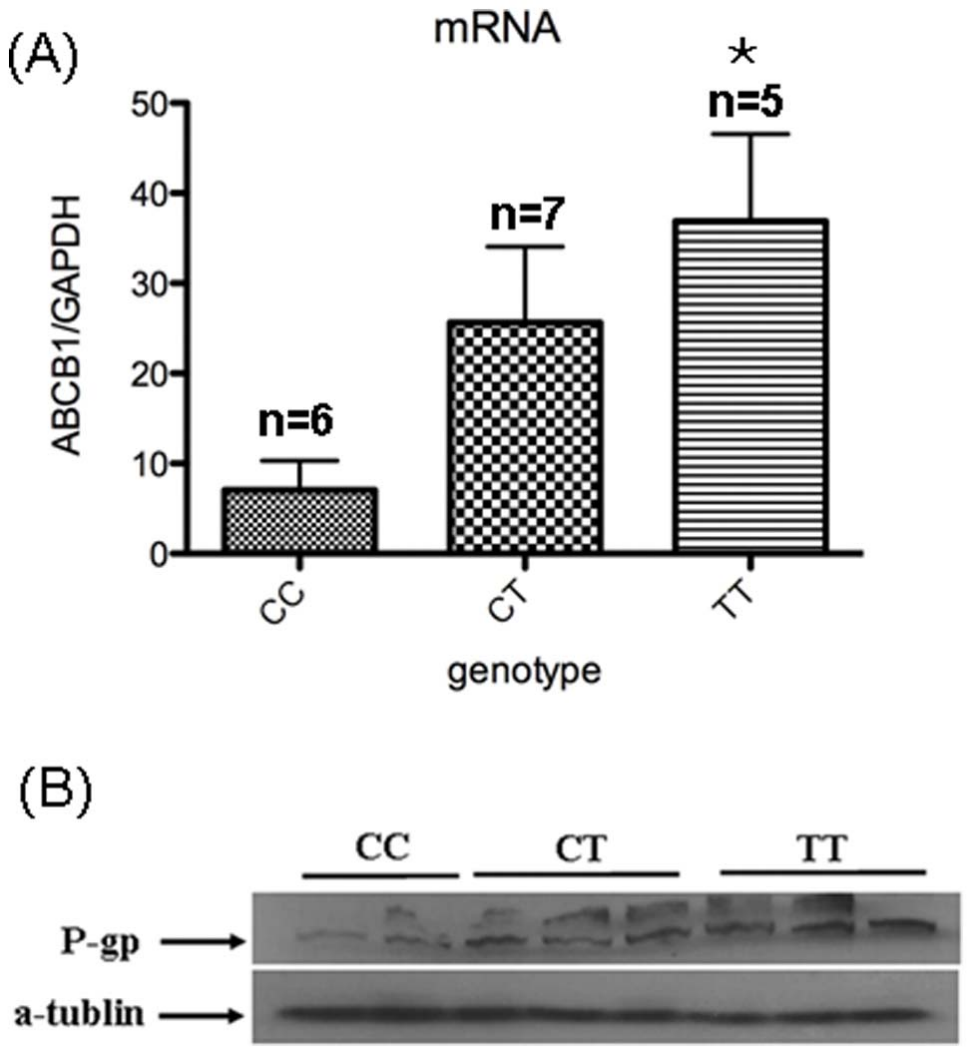
ABCB1 genotype-dependent mRNA and protein expression in the human placenta. (A) ABCB1 mRNA expression was determined by real-time qPCR in human placenta (n = 5–7) from women with different genotype of C3435T polymorphism. Data are presented as mean±SEM. Differences among the three genotypes were determined by one-way ANOVA followed by a Student-Newman-Keuls multiple comparison post hoc tests. The mRNA expression of the TT genotype was significantly higher than that of the CC genotype (* P = 0.03). (B) Western blot of P-gp expression in human placenta. Compared with the CC/CT genotype, higher P-glycoprotein expression was observed for the TT genotype.

The genotype distribution of C3435T polymorphism for the 35 umbilical cords were listed as follows: CC: 10(28.6%), CT: 20(57.1%), TT: 5(14.3%). After matched with maternal age for TT group (27–33 years old), 6 placentas in CC genotype group and 7 placentas in CT genotype group were recruited for mRNA and protein analyses. Clinical data of mothers and neonates stratified by C3435T genotype were illustrated in table 6. There were no significant differences in maternal age, BMI, gestational duration, gravity, placenta weight, neonatal weight and length among three genotype groups. As shown in Figure 1 (A), the ABCB1 mRNA expression of the TT genotype was significantly higher than that of the CC genotype (P = 0.03). Meanwhile, compared with TT genotype, lower P-glycoprotein expression was observed for the CC/CT genotype (Figure 1 B).

**Table 6:** Clinical data of mothers and neonates stratified by C3435T genotype.

*Characteristics*	*CC(n = 6)*	*CT(n = 7)*	*TT(n = 5)*	P value a
Maternal age (years)	30.50±2.59	29.57±1.62	30.60±2.70	0.392
Body mass index(kg/m^2^)^b^	28.89±4.68	26.34±4.34	27.35±0.80	0.609
Gestational duration(weeks)	39.67±0.82	39.57±1.72	38.80±1.79	0.595
Gravity	2.33±1.63	2.29±1.60	1.80±1.10	0.813
Neonatal gender (Female)	6(5)	7(5)	5(3)	–
Neonatal birth weight(g)	3375.00±170.97	3306.43±321.30	3269.00±419.71	0.849
Neonatal birth length(cm)	50.17±0.41	49.86±1.57	49.60±1.52	0.769
Placenta weight (g)	570.00±66.33	590.00±47.61	591.40±13.22	0.709

a Differences among three genotypes were determined by one-way ANOVA followed by a Student-Newman-Keuls multiple comparison post hoc tests.

b Body mass index(BMI) was calculated as weight divided by height (m) squared.

## Discussion

Results from our hospital-based study add to the literature proving that periconceptionally occupational exposure of the mothers to phthalates and alkylphenolic compounds increases the risk of CHDs, especially for septal defects, even after adjustment for confounding factors. Consistent with most of the reported results, the association in our study is weak. However, it provides supportive evidence that children have different susceptibilities to the adverse effects of toxic agents, thereby implying the inheritance of some particular genotypes might modulate the effect.

Highly expressed early in fetal life and placenta, ABCB1 appears to be the most predominant transporter in the human placenta [7], [8], [9], [10], [11], [14]. Several studies have revealed that P-gp exerted a protective role for the fetus by restricting the penetration of toxicants from the maternal circulation to the fetal compartment and the presence of C3435T polymorphism affects its activity [9], [14], [15], [16], [17], [21], [22], [23], which in turn may impair the efflux of toxicants. Nevertheless, data on maternal toxicants exposure and the role of this polymorphism in the pathogenesis of congenital malformations are insufficient. A study conducted by Bliek BJ and colleagues in 2009 showed a significant association between this polymorphism and cleft lip/palate [31]. However, a recent study failed to confirm it [32]. As far as we know, the current study is the first one to examine the role of ABCB1 gene C3435T polymorphism in the etiology of CHD related to maternal toxicants exposure. Our data uncovered the joint effects of the ABCB1 gene C3435T polymorphism and maternal periconceptional phthalates and/or alkylphenolic compounds exposure(s) on CHD risk in a Han Chinese population, suggesting that children who carry the CC/CT genotype and have periconceptionally exposed to phthalates and/or alkylphenolic compounds could suffer an almost 3.5-fold risk of having CHD than non-exposed children with TT genotype (adjusted OR: 3.5, 95%CI: 1.5–7.9, P-value: 0.003). Particularly, the OR changed to 4.4 for septal defects (adjusted OR: 4.4, 95%CI: 1.8–10.9, P-value: 0.001).

The C3435T SNP does not locate in any coding or regulatory regions of the ABCB1 gene; and consensus on the effect of this SNP on P-gp expression and transport activity in the placenta remains obscure at present. A study conducted by Rahi M and collegues in 2008 revealed that T alleles were associated with a higher placental P-gp protein level compared with the C alleles, but this difference did not have any consequences on P-gp-mediated placental transfer of saquinavir [33]. In contrast, Hemauer and colleagues in 2009 reported that, in human term placenta, the C3435T variant was associated with a 16% reduction in the P-gp protein level. However, placental P-gp activity did not seem to correlate with its expression level because homozygote for T alleles was found to have a 47% increase in P-gp mediated ATP-dependent uptake of the prototypic substrate [^3^H]-paclitaxel into vesicles prepared from placental brush border membranes [23]. Evidence suggests that P-gp locates in the maternal-facing apical membrane of the syncytiotrophoblast, which is of fetal origin. However, in the above two studies, genotype was determined by DNA isolated from whole placental tissue, which comprises both maternal and fetal cell populations and therefore might hamper a clear genotype-phenotype correlation. This shortcoming precludes any clear conclusion being made from these data. For the reasons mentioned above, umbilical cords were collected for fetal genotyping in the present study. Our preliminary results suggested that the ABCB1 mRNA expression level of the TT genotype was significantly higher than that of the CC genotype. Meanwhile, compared with TT genotype, lower P-glycoprotein expression was observed for the CC/CT genotype. These findings might explain higher risk of CHDs for C carriers identified in our study. However, because of limited placenta sample size and lack of measurement of P-gp transport activity, well-designed investigations with a large sample size on the relationship between this SNP and not only P-gp protein or mRNA expression, but most importantly, P-gp transport activity in placenta are needed in future works to address this issue, especially in Han Chinese population.

Some limitations must be considered when the results of this study are interpreted. Firstly, occupational exposure assessment in this study was based on a JEM, which was originally developed for jobs in Western Europe [24]. Additional problems may arise when the JEM is applied to populations different from the original target. However, we have taken some efforts to make adjustments for the difference in exposure probabilities between countries. As stated in the newly updated JEM in 2009, the exposure scenarios that describe the reasoning for the exposure probability scores are meant to be helpful for improving the JEM and for making adjustments to account for differences in exposure probabilities over time and between countries. It also pointed out that task specific information from questionnaires can be used to verify the exposure scenarios for the subjects in a specific study population [25]. Thus when we conducted the questionnaires, not only job titles but also the information of their main tasks and duties were collected. The original JEM only served as a starting point or reference material, three Chinese industrial hygienists from School of Public Health, Sichuan University, were asked to make adjustments for exposure assessment based on their knowledge of tasks and work environment of various occupations in China. For example, we adjusted the exposure probability scores for the scenario “working with sheep dipping pesticides”, as sheep dipping is much less common in China than in the UK. Also, all workers with the job around a farm (eg, feeding farm animals), pesticide exposure were less likely. Finally, exposure probability scores were adjusted based on the consensus judgments of these three experts. As shown in the literature, with such adjustments, different country specific JEMs have been used in various studies [34], [35], [36]. Therefore, we believe that the adjusted JEM could be appropriate for exposure assessment in the present study.

A limitation of the JEM is that it can not reflect intensity, or frequency of exposure. We tried to reduce the misclassification by assessing exposure based on both job titles and description of the work tasks. The outcome of the matrix, however, must be interpreted cautiously as exposure probabilities are only a crude measure of exposure.

Secondly, our study only included the newly house decoration as an environmental exposure. Other background exposures were not considered, the reasons are as follows: firstly, we admitted that for many chemicals, most people experience a certain level of exposure through diet, environment, or widely used consumer products (such as air pollution), however, the level of exposure to chemicals within occupations scored by the JEM is generally much higher than background exposure through diet and environment, and two studies have confirmed that higher concentration of phthalates and alkylphenolic compounds was present in newly decorated apartments [26], [27]. Furthermore, it is unlikely that those background exposures with a high prevalence are associated with congenital heart defects with a low prevalence. Thus, those background exposures will most unlikely confound the associations found in the present study. Even so, since we did not assess background exposure, which may have contributed to the unexplained variance in our results, and therefore residual confounding cannot be completely ruled out.

Thirdly, for phthalates and alkylphenolic compounds, it is still unknown whether they are P-gp substrates. Thus the detrimental effect of P-gp might be over- or underestimated. However, accumulating evidence suggests that P-gp is able to transport an extremely wide variety of chemically and structurally compounds. P-gp substrates are usually organic molecules ranging in size from about 200 Da to almost 1900 Da [8], [10], [14], [37], [38]. Therefore, this might be not an issue.

Recall bias is inevitably one of the pitfalls of case-control designs because of the time delay from delivery to interview. However, evidence is emerging that its role is rather small, and almost no differential in case-control studies focusing on congenital malformations [39]. In addition, the foundation of the occupational exposure assessment in the present study was based on the JEM, which is generally less influenced by recall bias as they are solely based on maternal reports job titles and tasks description, rather than the details of her potential on-the-job solvent exposures. Moreover, both case and control mothers did not know the outcomes of exposure assessment when they reported their job titles and tasks. Furthermore, although the questionnaire was filled-out 3 years after child birth; the work history in general is recalled quite easily and the job characteristics were available in 99% of the parents in the present study. In addition, masking was used in this investigation, and all interviewers were uniformly trained before the formal interview. Therefore, we believe the recall bias was minimal in the present study.

The last but not the least, an undoubted limitation of our study was the modest size of our population and the results of the separate CHD phenotypes should be further investigated in much larger data sets.

In spite of the limitations, we had tried our best to avoid the bias to a great extent and our results were adjusted by potential confounding factors. Furthermore, the preliminary results of the functional study also sustained the associations found in our study. Thus this significant association could not solely explained by recall bias or confounding by indications. As highlights of the present study, for the first time, our results suggested that maternal periconceptional exposures to phthalates and/or alkylphenolic compounds increase the risk of CHD/septal defects and fetal genotype with respect to ABCB1 gene is important in determining fetal susceptibility to CHDs. In conclusion, it is rather better to avoid exposures to phthalates and/or alkylphenolic compounds during the periconceptional period, especially for the fetal carriers of CC/CT genotypes of the ABCB1 gene.
